# Gastric Xanthoma: A Rare Case Report

**DOI:** 10.30699/ijp.2023.551854.2878

**Published:** 2023-02-01

**Authors:** Pummi Kumari, Ruchi Sinha, Nisha Khanna, Tanmoy Maji

**Affiliations:** 1 Department of Pathology, Lab medicine, All India Institute of Medical Science, Patna, Bihar, India; 2 Department of Gastroenterology, All India Institute of Medical Science, Patna, Bihar, India

**Keywords:** Altered lipid profile, Stomach, Xanthoma

## Abstract

Xanthomas are characterized by the presence of foamy lipid-laden macrophages. The gastrointestinal tract is an uncommon site for xanthoma, with the stomach being the most favored location. They have been associated with various premalignant and malignant conditions of the stomach. We present a case of a 21-year-old female patient with dyspepsia of 4 months duration. Her lipid profile was mildly altered. On upper gastrointestinal endoscopy, multiple discrete yellow patches were found in the antrum, diagnosed as gastric xanthoma on microscopy. Various published literature has emphasized the frequent association of gastric xanthomas with gastritis, gastric atrophy, intestinal metaplasia, and gastric cancer. Hence, there is a necessity for early recognition, treatment of any coexistent pathology, and close clinical follow-up.

## Introduction

Xanthomas, or xanthelasma, or lipid islands, are an uncommon non-neoplastic entity of the gastrointestinal (GI) tract, with the stomach being the most favored GI location (1). It is characterized by the accumulation of lipid-laden foamy macrophages in the mucosa. Various studies have reported the incidence of gastric xanthoma varying from 0.23% to 7% (1,2). Its prevalence is more frequent in east Asian countries compared to western countries (2,3). Gastric xanthoma has been reported in all age groups, but its incidence increases with age (4,5).

Histologically foamy macrophages of gastric xanthoma can mimic Whipple disease or malignancies like signet ring cell adenocarcinoma and clear cell type of carcinoid tumor (6,7).

Several studies have found a significant association of xanthoma with precancerous lesions of gastric mucosa like atrophy, Helicobacter pylori infection, and also with gastric cancer (8–11). It has the potential to be used as a predictive marker for early gastric cancer (10,12). So, despite being a benign entity, gastric xanthoma demands prompt diagnosis and close follow-up.

## Case Presentation

A 21-year-old female presented to All India Institute of Medical Sciences Patna, in 2021 gastroenterology OPD with dyspepsia (upper abdominal pain, burning sensation, bloating, and nausea) for 4 months. No other significant history or family history was noted. She had no addiction. Her physical examination was normal. Her routine laboratory investigation (complete hemogram, liver function test, and kidney function test) were within normal limits. 

**Fig. 1:**
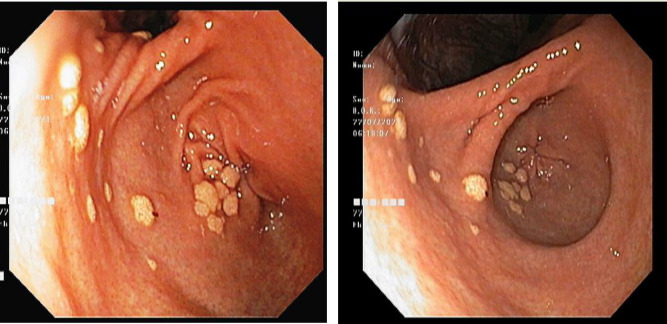
Multiple discrete yellowish-white plaques in the antrum of the stomach on upper GI endoscopy

An upper GI endoscopy revealed multiple discrete whitish-to-yellowish patches measuring less than 1 cm in size in the gastric antrum (Figure 1). No lesion was identified in the other parts of the stomach, esophagus, and duodenum on endoscopy. Based on these findings, a differential diagnosis of lymphangiectasia or candidiasis was suggested. Multiple biopsies were taken from the lesion and sent for histopathology.

**Fig. 2:**
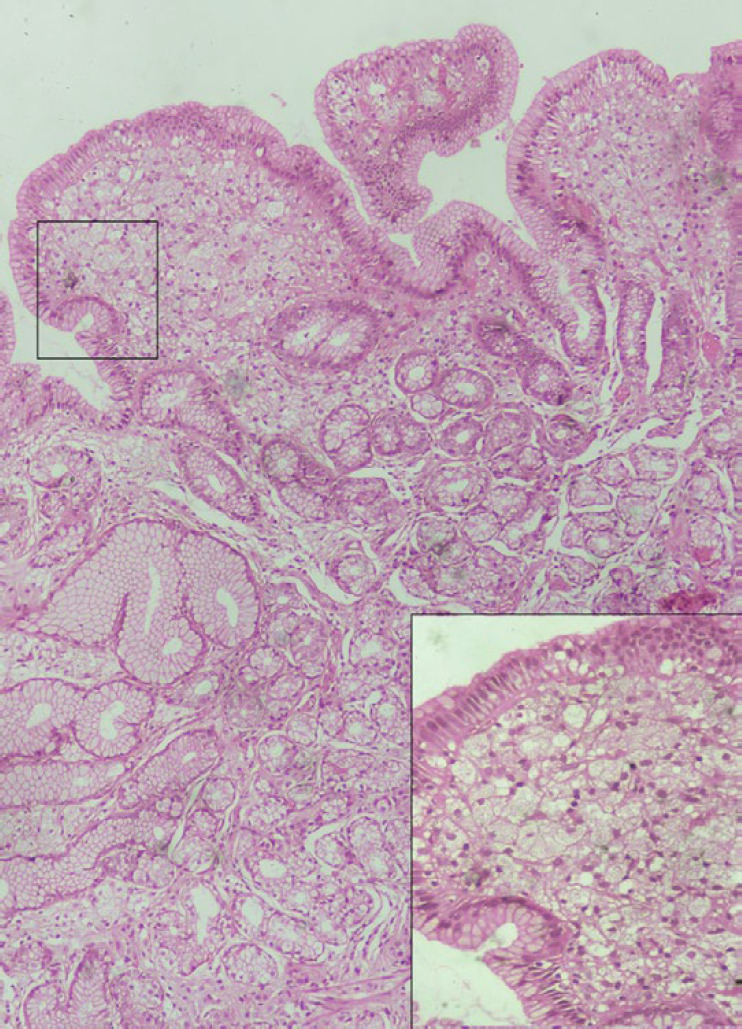
Widening of foveolae by sheets of xanthoma cells. (H&E; 10X). Inset: High power view showing xanthoma cells (H&E; 40X).

Microscopic examination of the gastric biopsy showed a widening of foveolae by sheets of polygonal cells with abundant foamy cytoplasm and bland central nucleus (Figure 2). However, no features of metaplasia or dysplasia were noted in the epithelial cells. Also, no dilated lymphatic channels, giant cells, or granuloma were seen in the biopsy tissue. Giemsa stain for Helicobacter pylori was negative. Ziehl- Neelsen (ZN) stain was also negative for acid-fast bacilli.

**Fig. 3:**
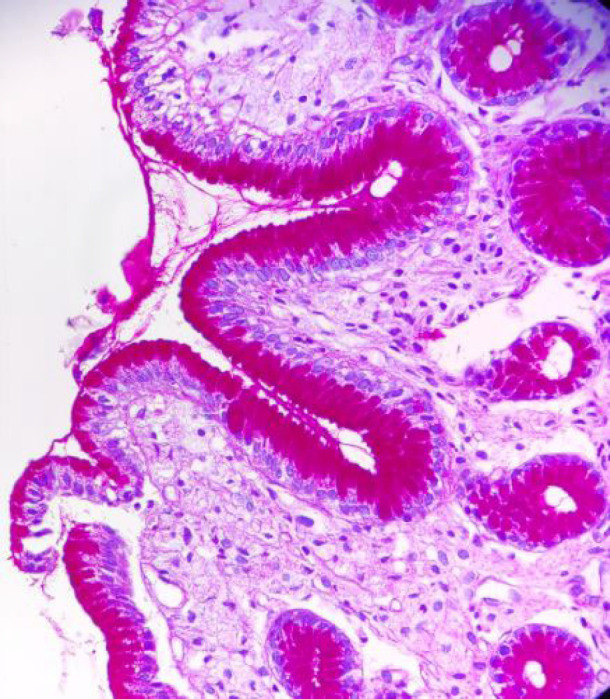
Xanthoma cells are PAS negative (40 X).

The foamy cells showed a negative reaction on Periodic acid –Schiff (PAS) staining (Figure 3). This ruled out fungal etiology and distinguished vacuolated macrophages from mucin-secreting adenocarcinoma cells. All these features suggested the diagnosis of Gastric xanthoma.

**Fig. 4:**
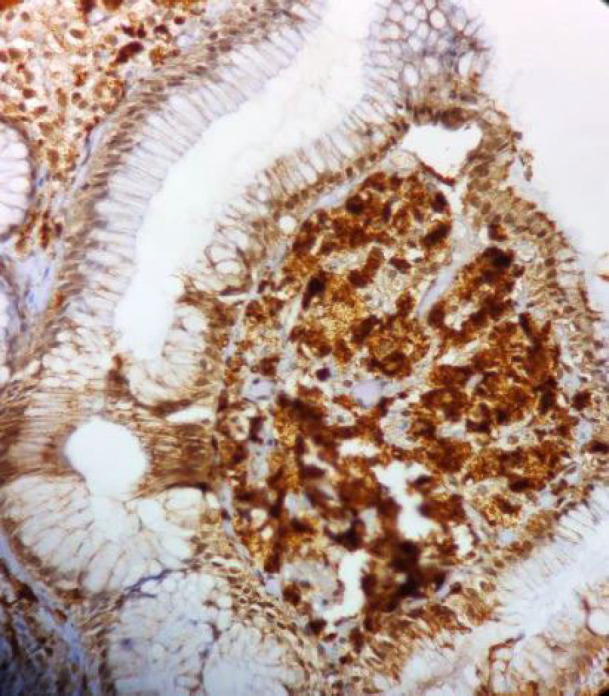
Immunohistochemical staining with CD68 showing diffuse cytoplasmic staining (40X)

Immunohistochemical (IHC) staining with CD 68 showed diffuse and robust cytoplasmic positivity in the foamy cells (Figure 4)confirming the histiocytic lineage of these cells.

Based on the above histopathologic, histochemical, and IHC findings, a diagnosis of gastric xanthoma was made.

Given the histopathological diagnosis of gastric xanthoma, a lipid profile was ordered, which showed raised cholesterol/HDL ratio (5.22) [normal <4.5] and LDL/HDL ratio (3.42) [normal <3]. Serum LDL level was on the upper normal limit (94 mg/dL) [normal <100 mg/dL]. Total serum cholesterol, triglyceride, and HDL were within normal limits.

## Discussion

Gastric xanthoma was first described by Orth in 1887 (13). It is found most commonly in the stomach, followed by the esophagus and duodenum, and rare cases have been reported in the colorectum (1). Cases of gastric xanthoma have been reported mainly in eastern countries, with incidence varying from 0.23-7% (1,2).

It is more common in the elderly but can have a wide range of ages at presentation (14,15). Clinically, gastric xanthomas tend to be asymptomatic, and if symptomatic, they are typically related to coexisting pathology (1). 

On upper GI endoscopy, gastric xanthoma presents as discrete single to multiple yellowish-white patches, most commonly in the antrum or pyloric region of the stomach (13). Endoscopically it resembles candidiasis, polyp, lymphangiectasia, Russell body gastritis, and Pseudoxanthoma elastic or gastric malignancy (3,16,17). Hence, gastric xanthoma is challenging to identify on endoscopy. The present case on upper GI endoscopy showed multiple discrete whitish-to-yellowish patches measuring less than 1cm in the stomach's antrum. 

Histologically, it consists of aggregates of foamy macrophages containing lipids in the upper lamina propria of the gastric mucosa. Microscopically, these cells can be confused with Whipple disease, mycobacterial infection, submucosal lipoma, signet ring cell adenocarcinoma, and clear cell type of carcinoid tumor (6,7). Special histochemical stains like PAS, ZN, and IHC staining with CD68 helps differentiate gastric xanthoma from the entities mentioned above and confirm its histiocytic origin. The xanthoma cells are PAS and ZN negative and show diffuse cytoplasmic CD68 positivity.

Although the pathogenesis of gastric xanthoma is yet not clear, it has been hypothesized that gastric mucosal injury has a role as gastric xanthoma is frequently associated with atrophic gastritis, intestinal metaplasia, hyperplastic polyp, and Helicobacter pylori infection (3). Gastric xanthoma may result from an inflammatory response toward gastric mucosa damage (2). 

Some previous literature has also documented dyslipidemia in gastric xanthoma (2). One possible explanation for this could be that, as in xanthoma, there is an accumulation of cholesterol, neutral fats, low-density lipoprotein (LDL), and oxidized LDL (18). This oxidized LDL may lead to increased release of oxygen free radicals. Diabetes, too, can predispose to gastric xanthoma due to increased free radical production (10). Free radicals are potent carcinogens that can cause DNA damage and lead to cancer development. In our case, the patient presented with a mildly deranged lipid profile with elevated serum cholesterol/HDL and LDL/HDL ratio. 

Several studies have shown a high prevalence rate of gastric xanthoma in gastric cancer patients. Kitamura *et al.* observed xanthoma in 72.5% of cases of gastric cancer with a higher prevalence of differentiated adenocarcinoma (12). Yamashita *et al.* have found the overall prevalence of gastric xanthoma to be 14.2% in gastric cancer patients (19). Sekikawa *et al.* found that the overwhelming majority of gastric carcinomas occurred in the same location as gastric xanthoma (10). All these factors suggest that gastric xanthoma can be considered a potential predictive marker for the presence of gastric cancer. 

## Conclusion

Gastric xanthoma is a benign collection of lipid-laden foamy macrophages, which morphologically mimics a variety of non-neoplastic and gastric lesions. This underlies the role of pathologists in careful evaluation and definite diagnosis. Microscopic examination is also important to rule out any coexisting gastric pathology using various special and immunohistochemical stains. The frequent association of gastric xanthomas with gastritis, gastric atrophy, intestinal metaplasia, and gastric cancer emphasis its recognition, treatment of any coexistent pathology, and close clinical follow-up.

## Conflict of Interest

There is no conflict of interest.

## Funding

None.

## References

[1] Gencosmanoglu R, Sen-Oran E, Kurtkaya-Yapicier O, Tozun N (2004). Xanthelasmas of the upper gastrointestinal tract. J Gastroenterol.

[2] Yi SY (2007 ). Dyslipidemia and H pylori in gastric xanthomatosis. World Journal of Gastroenterology.

[3] Basyigit S, Kefeli A, Asilturk Z, Sapmaz F, Aktas B (2015). Gastric Xanthoma: A Review of the Literature. Shiraz E-Med J.

[4] Naito M, Miura S, Funaki C, Tateishi T, Kuzuya F (1991). [Gastric xanthomas in the elderly]. Nihon Ronen Igakkai Zasshi.

[5] Moretó M, Ojembarrena E, Zaballa M, Tánago JG, Ibáñez S, Setién F (1985). Retrospective endoscopic analysis of gastric xanthelasma in the non-operated stomach. Endoscopy.

[6] Luk IS, Bhuta S, Lewin KJ (1997). Clear cell carcinoid tumor of the stomach A variant mimicking gastric xanthelasma. Arch Pathol Lab Med.

[7] Jain S, Mahajan V, Kumar M (2015). Xanthelasma of the stomach--A rare pseudotumor. Trop Gastroenterol.

[8] Isomoto H, Mizuta Y, Inoue K, Matsuo T, Hayakawa T, Miyazaki M (1999). A close relationship between Helicobacter pylori infection and gastric xanthoma. Scand J Gastroenterol.

[9] Liu C-X, Shen Y-Y, Shi N, Hu Y-B, Jia X-F, Zhou C-J (2018). Correlation of endoscopic images and histological findings of a high grade dysplasia developed in a gastric xanthoma. Int J Clin Exp Pathol.

[10] Sekikawa A, Fukui H, Sada R, Fukuhara M, Marui S, Tanke G (2016). Gastric atrophy and xanthelasma are markers for predicting the development of early gastric cancer. J Gastroenterol.

[11] Shibukawa N, Ouchi S, Wakamatsu S, Wakahara Y, Kaneko A (2019). Gastric Xanthoma Is a Predictive Marker for Early Gastric Cancer Detected after Helicobacter pylori Eradication. Intern Med.

[12] Kitamura S, Muguruma N, Okamoto K, Tanahashi T, Fukuya A, Tanaka K (2017). Clinicopathological Assessment of Gastric Xanthoma as Potential Predictive Marker of Gastric Cancer. Digestion.

[13] Khachaturian T, Dinning JP, Earnest DL (1998). Gastric xanthelasma in a patient after partial gastrectomy. Am J Gastroenterol.

[14] Dhakal M, Dhakal OP, Bhandari D, Gupta A (2013). Gastric xanthelasma: an unusual endoscopic finding. BMJ Case Rep..

[15] Andrejić BM, Božanić SV, Solajić NS, Djolai MA, Levakov AM (2012). Xanthomas of the stomach: a report of two cases. Bosn J Basic Med Sci.

[16] Cocco AE, Grayer DI, Walker BA, Martyn LJ (1969). The stomach in pseudoxanthoma elasticum. JAMA.

[17] Yoon JB, Lee TY, Lee JS, Yoon JM, Jang SW, Kim MJ (2012). Two Cases of Russell Body Gastritis Treated by Helicobacter pylori Eradication. Clin Endosc.

[18] Kaiserling E, Heinle H, Itabe H, Takano T, Remmele W (1996). Lipid islands in human gastric mucosa: morphological and immunohistochemical findings. Gastroenterology.

[19] Yamashita K, Suzuki R, Kubo T, Onodera K, Iida T, Saito M (2019). Gastric Xanthomas and Fundic Gland Polyps as Endoscopic Risk Indicators of Gastric Cancer. Gut Liver.

